# A Narrative Review of High Throughput Wastewater Sample Processing for Infectious Disease Surveillance: Challenges, Progress, and Future Opportunities

**DOI:** 10.3390/ijerph21111432

**Published:** 2024-10-29

**Authors:** Bhuvanesh Kumar Shanmugam, Maryam Alqaydi, Degan Abdisalam, Monika Shukla, Helio Santos, Ranya Samour, Lawrence Petalidis, Charles Matthew Oliver, Grzegorz Brudecki, Samara Bin Salem, Wael Elamin

**Affiliations:** 1 RASID Laboratory, M42 Healthcare, Abu Dhabi P.O. Box 4200, United Arab Emirates; 2 Mubadala Health-Dubai, Dubai P.O. Box 26699, United Arab Emirates; 3 Abu Dhabi Quality and Conformity Council (ADQCC), Abu Dhabi P.O. Box 2282, United Arab Emirates

**Keywords:** wastewater-based epidemiology (WBE), operational challenges, disease surveillance, pandemic preparedness, automation, artificial intelligence

## Abstract

During the recent COVID-19 pandemic, wastewater-based epidemiological (WBE) surveillance played a crucial role in evaluating infection rates, analyzing variants, and identifying hot spots in a community. This expanded the possibilities for using wastewater to monitor the prevalence of infectious diseases. The full potential of WBE remains hindered by several factors, such as a lack of information on the survival of pathogens in sewage, heterogenicity of wastewater matrices, inconsistent sampling practices, lack of standard test methods, and variable sensitivity of analytical techniques. In this study, we review the aforementioned challenges, cost implications, process automation, and prospects of WBE for full-fledged wastewater-based community health screening. A comprehensive literature survey was conducted using relevant keywords, and peer reviewed articles pertinent to our research focus were selected for this review with the aim of serving as a reference for research related to wastewater monitoring for early epidemic detection.

## 1. Introduction

**Figure 1:**
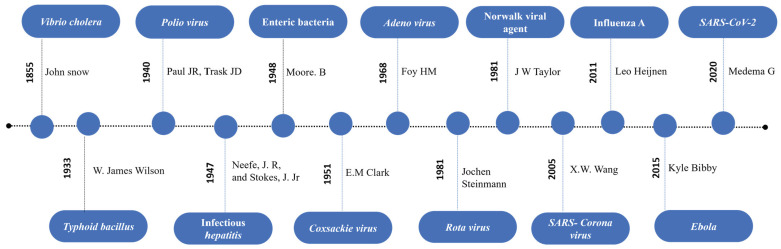
Chronological representation of major milestones of wastewater-based epidemiology [17,18,19,20,21,22,23,24,25,26,27].

Wastewater is mostly considered a source of environmental pollution, and most established tests are performed to determine the pollutant load and to assess the treatment efficacy. However, it also provides useful information including biological traces, lifestyle drugs [1,2], and a variety of disease markers that threaten our well-being [3]. In 1854, John Snow first documented the waterborne transmission of Cholera and was able to identify the exact source [4]. Since then, epidemiologists studied water to understand how diseases spread and how they can be tracked to contain disease outbreaks, such as polio [5] and hepatitis A virus (HAV) [6]. A study of influenza in wastewater was performed during the H1N1 (swine) influenza virus outbreak. While influenza A viruses were found in sewage, the H1N1 pandemic virus was not detected in wastewater. This study confirmed the ability of quantitative real-time reverse transcription polymerase chain reaction (qRT-PCR) testing to detect influenza viruses in sewage samples [7]. Similarly, *Aeromonas hydrophilia*, *E. coli*, [8] *Enterococcus* spp., *Staphylococcus* spp., and coliform bacteria [9] were successfully detected from wastewater using a PCR-based method. In the following years, Enteric viruses such as noroviruses (NoVs), sapovirus (SV), hepatitis A and E viruses, adenoviruses (AdVs), rotaviruses (RVs), astroviruses (AstVs) [10,11,12], and respiratory viruses [13] were detected from wastewater samples. Recent reports of Zika and Ebola virus in wastewater suggest that these viruses could also be potential targets for continuous wastewater surveillance [14,15,16]. The milestones and pathogens reported from wastewater are summarized in Figure 1 below.

Recently, WBE has been widely used to monitor severe acute respiratory syndrome coronavirus 2 (SARS-CoV-2) infections [27,28]. Several countries deployed wastewater-based surveillance for SARS-CoV-2, as this method overcomes limitations of clinical surveillance, helps screening both symptomatic and asymptomatic individuals, and is more economical. Table 1 provides a detailed list of countries conducting wastewater surveillance for pathogens, along with links to their respective data dashboards. WBE has the potential to complement clinical infectious disease surveillance and can act as an early warning system for disease outbreaks [29]. This narrative review discusses the challenges faced in wastewater surveillance for infectious disease and recent advancements and explores prospects of full-fledged wastewater-based communal screening.

A comprehensive literature review was conducted to gather relevant data for this study. Key search terms included “wastewater surveillance pathogen”, “public health”, “sampling for wastewater monitoring”, and “automation in wastewater surveillance” and were used across databases including PubMed, Pro Scopus, ScienceDirect, and JSTOR. Articles were selected by two independent reviewers (WE and BKS) based on their relevance and connection to the study’s focus. Only peer reviewed and published articles were considered in this review.

### 1.1. Advances in Understanding the Key Factors for Wastewater-Based Epidemiology

#### 1.1.1. Determining the Scale of Sampling

Traditionally, sampling sites and scales are chosen based on high-risk or vulnerable locations identified by epidemiologists. Choosing a suitable sampling technique and scale for wastewater analysis is crucial for obtaining a true representation [33]. Each sampling scale or site has its own merits and disadvantages. Screening at an inlet of a centralized wastewater treatment facility helps understand the disease dynamics of the catchment area. However, it fails to accurately specify the hotspot of infection (outbreak region), and a detailed map and a well-established sewer network is needed to facilitate narrowing down the areas of interest and enable a more accurate interpretation [34]. Alternatively, samples collected from sewer line manholes can provide geographically more precise data [35]. The tagging of manholes with geographical information (GIS) and access to population data can improve the correlation of results. This approach requires multiple sampling points to cover a region, and accessing a manhole located in a high-traffic location requires special permission from authorities. Other sampling sites, such as those from closed communities, are more precise, with most studies and simulations performed in such closed setups aiming toward a better understanding and correlation with clinical testing [36,37,38,39]. Overall, sampling at a centralized wastewater treatment facility is more practical and economical for routine analysis; upon identification of a biomarker or agent of interest, the source can be further investigated upstream and can be located via more intense sampling at the level of neighborhood sewer manholes, blocks, and buildings, followed by clinical surveillance for the implementation of mitigation efforts.

Wastewater samples from cruise ships and long-haul passenger aircrafts have also been studied in recent times [40,41]. As air travel is one of the prime means of infectious disease spreading worldwide, the probability of detecting pathogens from aircraft samples relies on lavatory usage. According to survey results conducted by Davey. L Jones et al. [42], approximately 13% of passengers defecate during short-haul flights, and it rises to 36% for long-haul flights. Wastewater collected from the aircraft lavatories provided an earlier detection when compared to respiratory swab testing on 30% of travelers for SARS-CoV-2 screening [43].

#### 1.1.2. Sampling Techniques Adopted for Wastewater-Based Surveillance

WBE necessitates standardization in several aspects, including the selection of appropriate sampling techniques. Several attempts have been made to develop a simple, yet efficient sampling method to capture the entire microbiome of wastewater [44,45]. The three widely studied sampling methods are grab sampling (sample collected at a preset timing) from the source, composite sampling using an autosampler (a composite sample is made up of aliquots that are collected over a period, often 24 h), and passive sampling using a membrane attached to a suitable support (deployment of a device with a special membrane that interacts/attracts the biomarker present in the wastewater). In most cases, a grab sampling approach is used to cover larger populations (~100,000 to 300,000), while a composite sampling approach is employed for smaller catchment populations.

#### 1.1.3. Comparison of Different Sampling Methods—Passive Sampling

Passive sampling is achieved by exposing a collecting medium to wastewater, thereby enabling the exchange of analytes between the medium and the sample. Rafiee et al. (2021) [46] compared different sampling methods and suggested that the passive sampling by Moore [21,47] is equally efficient as composite sampling for SARS-CoV-2 virus detection and variant profiling in sewage [48,49]. Similarly, Schang et al. (2021) [50] reported that passive and composite sample methods are more effective at providing meaningful results for catchment areas [51]. Recently, different passive sampling devices have been designed and tested for wastewater. Melissa et al. (2022) [52], used a torpedo-style 3D-printed passive sampler device containing cotton swabs and an electronegative filter membrane and reported greater sensitivity than grab and composite (autosampler) sampling [53]. Acer et al. (2022) [54] attempted a passive sampling approach using tampons made from rayon with a polyester string and showed a positive correlation between wastewater viral load and positive caseload in the sampling region [55]. In a similar study, Michael et al. (2024) [56] attempted passive sampling using cost-effective sorption materials, such as cheesecloths, gauze swabs, electronegative filters, glass wool, and tampons housed in a torpedo-style setup, compared with standard composite sampling. Their research aimed to detect SARS-CoV-2, influenza A and B viruses, crAssphage, and human AdV. The authors concluded that the selected passive sampling method/material was more effective for detecting other viruses studied than for SARS-CoV-2. Bivin et al. (2022) [44] undertook a comprehensive review of passive sampling and demonstrated a heterogeneous correlation with concentrations from paired composite samples of individual studies. Further studies have recommended that the usage of electronegative membranes over nitrocellulose serves effectively for monitoring SARS-CoV-2 viruses. Although passive samples are comparable to other sampling methods, the main drawbacks of passive sampling are that the medium tends to saturate with time and the specificity of material used toward the pathogen of interest, and the results acquired are mostly used for qualitative or semi-quantitative analysis.

#### 1.1.4. Composite Sampling and Grab Sampling

A composite sample is a single sample volume made up of aliquots, collected over a predetermined amount of time. The method is further divided into time-dependent and flow-dependent applications, where the aliquot volume is altered in a way that is proportional to the wastewater flow, or the sample time is altered based on the application. Ahmed et al. (2021) [57] demonstrated in their study that 24 h composite samples offered increased sensitivity and decreased variability compared to 1 h composite samples when monitoring wastewater for pathogenic viruses with low infection rates within a community. In contrast, a study conducted by Augusto et al. (2022) [58] on SARS-CoV-2 detection showed that grab samples collected during peak flow matches the mean value of the 24 h composite samples. The release of biomarkers in wastewater changes throughout the day due to diurnal activity. Therefore, obtaining a grab sample at a predetermined time is likely to capture most of the predominant pathogens from wastewater. However, this approach might fail to detect pathogens of low concentration. Although the grab sample method is easy to operate but prone to variability in results, the composite sample method provides stable and accurate data. However, its prohibitive cost and operational difficulties make it unsuitable for small-scale studies [59]. Furthermore, for WBE applications, the usage of 24 h composite samples and passive sampling techniques with electronegative mesh are recommended, as a more precise method to provide accurate results. Both sampling methods are reliable, whereas passive sampling is more practical, efficient, and cost-effective. Table 2 provides a summary of various sampling methods used for wastewater surveillance.

#### 1.1.5. Shelf Life and the Importance of Turnaround Time

The persistence of bacteria in wastewater is affected by several factors such as temperature, dissolved oxygen, pH, nutrient availability, and salinity [60]. Bacteria tends to survive longer in wastewater than in surface water under similar storage conditions [61]. Shuxin Zhang et al. (2023) [62] investigated the decay of two gastrointestinal pathogens, *Campylobacter jejuni* and *Campylobacter coli*, in wastewater. The decay under different sewer conditions was investigated in a reactor, and the result signifies that at a higher spike concentration (10^−6^/mL), about 9.76% recovery was achieved after 36 h. Considerable variations in the recovery were also observed within the same genus. Unlike bacteria, viruses need a host cell to multiply, and therefore the viral concentration present in wastewater would be proportional to the concentrations excreted by the corresponding population.

Diversity in the wastewater matrix, dilution effects, and the presence of reactive chemicals may affect the viral particle concentration and integrity [63]. Thus, the interpretation of results requires an understanding of the stability of the virus in wastewater. The effect of temperature on SARS-CoV-2 RNA detection in wastewater surveillance has not been considered in most reported studies [64]. Non-enveloped viruses have been reported to be more stable at varying temperatures than enveloped viruses [65,66]. In winter months, the viruses are more stable in wastewater and can be detected for up to 100 h, whereas in summer months, it is reduced to 20 h. These temperature-related variations were comparatively lower in locations with lower water temperatures, as the viral RNA was preserved for a longer duration [67]. Lisa et al. (2009) [68] evaluated the survival of two surrogates of coronaviruses, transmissible gastroenteritis (TGEV), and mouse hepatitis (MHV). At 4 °C, there was a <1 log10 decrease in infectivity for both viruses after four weeks. In pasteurized sewage, a 99% reduction was reported in 9 days for TGEV and 7 days for MHV. In a study conducted by Markt et al. in 2021 [69], the author compared the impact of storing 24 h composite wastewater samples at temperatures of −20 °C and ≤4 °C and observed that the storage of wastewater for up to 9 days at 4 °C did not have a significant impact on the number of detectable SARS-CoV-2 fragments. Similarly, Ahmed et al. (2020) [70] compared different storage conditions and different matrix effects and reported a decay rate of approximately 8% per day at 4 °C. The impact of storing SARS-CoV-2 in wastewater sludge at varying storage temperatures (4 °C, −2 °C, and −80 °C) was studied by Simpson et al. (2021) [71]. The author observed no change in concentration by 7 to 8 days when stored at 4 °C, and by 35 to 122 days, about a 60% reduction was observed. The author further reports that SARS-CoV 2 RNA in sludge is more stable in a freeze–thaw cycle than in a liquid effluent. Bonnie et al. (2022) [72] reported a seven-fold decrease in viral load when stored at−80 °C for 92 days compared to without freezing, and the author further observed that storage in a glycine-releasing buffer resulted in four-fold inhibitions. Attempts were made to filter the sample and store the filter paper containing virus particles; at −80 °C, this procedure had no significant loss in viral concentration for up to a month [73]. Several studies have used population biomarkers in wastewater to normalize the pathogen load [74,75]. Normalizing SARS-CoV-2 RNA concentrations by concentrations of PMMoV RNA, an endogenous wastewater virus, can correct for changes during storage, as the effect of storage parameters on PMMoV RNA is like that on SARS-CoV-2 RNA [76]. Finding a suitable surrogate for all other circulating pathogens is yet to be studied. In the case of samples collected from aircrafts, the pathogens are usually inactive as modern aircraft lavatory storage tanks are preloaded with bactericidal and viricidal agents to prevent disease spread. It is advisable to acquire an understanding of the target organism’s shelf life and the complexity of the wastewater before sample analysis. Based on the literature, a sample should be processed as soon as it reaches the laboratory. Under unforeseen circumstances, wastewater samples can be stored at 4 °C and the results should be adjusted based on the delay in analysis.

### 1.2. Advancements in Analytical Techniques for Detection of Pathogen from Wastewater

#### 1.2.1. PCR Based Detection Method

The identification and characterization of pathogenic microbes from wastewater microbiomes pose several challenges by classical plating and enumeration methods. The process is even more cumbersome for virus detection (e.g., polio), as the wastewater sample must undergo a high-volume chemical concentration method (PEG/Dextran) followed by cell line studies and then molecular typing for the positive samples [77]. The process of culturing viruses requires class 3 facilities and highly trained personnel to perform the analysis. Molecular methods circumvent these challenges and have made it possible to analyze and categorize the diversity of hazardous microorganisms. Nucleic Acid Amplification Tests (NAATs) are useful for detecting pathogens in wastewater samples [78]. A Spanish study demonstrated the detection of SARS-CoV-2 in wastewater while patients’ nasal swab PCR tests produced a negative result [79]. This has led to the acceleration of molecular technology as a method of choice for wastewater analysis [80]. There has been a combination of new detection tools utilized for the quantification and analysis of various gene targets to improve the sensitivity and accuracy of pathogen detection, which has led to the discovery of new surveillance opportunities [81]. However, it is important to consider the differences in PCR efficiencies and how this can significantly affect the quantification accuracy and the limit of detection [82]. The sensitivity of different PCR techniques is summarized in Table 3 and the pathogens identified in wastewater through various PCR analysis techniques are shown in Table 4.

Over the last few years, digital PCR (dPCR) has grown in popularity, especially for wastewater surveillance [84]. The principle of dPCR is to divide the sample into several independent partitions so that each contains few or no target sequences. Then, the dispersion of the target sequences in the partitions can be approximated using a Poisson distribution [115]. The ratio of the positive distributions (presence of fluorescence) to the total number makes it possible to determine the concentration of the target in the sample [80]. In a study made by Flood et al. (2021) [116], the authors revealed that the observed coefficient of variation (CV) for RT qPCR measures were much larger than the dPCR results for the absolute measurement of SARS-CoV-2 genetic markers in various wastewater matrices. In studies conducted by W Ahmed et al. (2022) [83,99], it was observed that untreated effluent from aircraft demonstrated the potential of dPCR to detect a higher number of positive samples compared to qPCR. Additionally the limit of detection using dPCR were approximately 2–5 times lower than those of observed with RT-qPCR. A cheaper and faster alternative method to dPCR is the NESTED PCR; this method was designed to improve the sensitivity and specificity of the reaction. It involves two sets of primers instead of one in two successive PCR reactions, as this improves the sensitivity and specificity. The advantage of this method is that it provides an exceptionally low probability of nonspecific amplification [117].

Another alternative technique that amplifies DNA with high specificity is the loop-mediated isothermal amplification (LAMP) technique. This technique has some advantages over qPCR and is used often in diagnostics [118]. LAMP PCR is one of the quickest, as the whole amplification can be performed under one set temperature, whereas PCR requires several cycles of different temperatures. Tumino et al. (2020) [119] compared the LAMP assay to a well-established TaqMan real-time PCR method for the detection of Mycoplasma agalactiae and showed that the LAMP assay was faster and more sensitive than the real-time PCR method (90% vs. 77%). Even though the LAMP-based method has a high sensitivity, its performance is affected when the infection prevalence is low. In areas where the infection number is low, the detection limit of 10 copies/25 µL may be a limitation. However, there were some noticeable improvements when the initial sample volume was increased from 1 to 5 µL [86]. Nonetheless, qualitative analysis now makes the most use of the RT-LAMP technique. As a result, its application is restricted to target detection and cannot offer information on the viral load. Nevertheless, as an early warning system, RT-LAMP is considered an ideal technique, as it provides information about the presence of SARS-CoV-2 in wastewater [120]. JE Ongerth et al. (2021) [121] used RT-LAMP in a quantitative application for the detection of SARS-CoV-2 in raw sewage. Primer sets targeting ORF1a, E- and N-gene regions were chosen to test for method performance characteristics for SARS-CoV-2 detection in the raw sewage of samples from a municipal sewage system serving >600,000 people in Australia; although the viral quantification was not consistent, it detected the virus in all the samples from each of the three independent interceptors near the treatment terminus. Haorui Cao et al. (2022) [122] developed a point-of-use detection method for SARS-CoV-2 using CRISPR/Cas12a and RT-LAMP in a paper device. The author reported an analytical sensitivity of 97.7% and an 82% semiquantitative accuracy. More recently, a high throughput microfluidic quantitative PCR system (HT-qPCR) was used to analyze wastewater for the presence of multiple pathogens and antibiotic resistance genes (ARGs) in a single run. By using this approach, more reactions can be achieved in a single plate, thereby reducing cost and improving TATs [123]. In another attempt, Rao et al. (2024) [124] used the PCR-Taqman array card (TAC) to detect infectious pathogens in wastewater; in this study, the author was able to quantify and analyze 35 pathogenic targets including bacteria, viruses, protozoa, and helminths simultaneously. Further, the author used two PCR platforms to validate the TAC, i.e., RT-qPCR quantstudio and dPCR QIAcuity four. In summary, PCR-based methods for the detection of pathogens from wastewater have immense potential in both in situ and ex situ testing, and they are constantly evolving to meet our testing capabilities.

#### 1.2.2. Wastewater Concentration for Enhanced Biomarker Detection

The sensitivity and accuracy of PCR techniques highly rely on the quality of the nucleic acid extracted from the wastewater. Unlike clinical samples, wastewater is diluted several thousand-fold and requires a concentration step prior to extraction to enhance the detection limit. Conventional concentration procedures involve polyethylene glycol (PEG), aqueous two-phase partitioning, ultracentrifugation, electronegative membrane filtration, ultrafiltration, salt-based precipitation, adsorption, and tangential flow filtration systems, and are laborious and time-consuming [125,126,127,128,129,130]. Recently, a high throughput automated sample concentration method has been suggested to reduce process fluctuation due to handling and reduce the labor-intensiveness of the protocol. Kevill. J.L. et al. (2022) [131] compared a PEG-based concentration method with precipitation (ammonium sulfate) and ultrafiltration using CP Select (Innovaprep) at different storage temperatures, turbidity, and surfactant concentrations for SARS-CoV-2 and fecal indicator virus (Assphage) recovery. Although all three chosen methods were found to be suitable, the PEG method yielded higher recovery due to longer exposure. After the precipitation and CP select methods, Wishlist CP recoveries are marginally better in quality and more consistent. This may be because most WBE studies target non-enveloped enteric viruses, whereas SARS-CoV-2 is an enveloped virus with a lipid layer that is sensitive to organic chemicals [132]. A single-step concentration method for SARS-CoV-2 using polyaluminum chloride precipitation was studied by Wehrendt et al. (2021) [133], where the authors report a 25-fold increase in sensitivity, and the precipitated sample was stable for a week at 4 °C. Similarly, Katayama et al. (2023) [134] used coagulation using poly aluminum chloride and digestion using proteinase K followed by magnetic bead-based extraction (COPMAN) for the extraction of SARS-CoV-2 from wastewater. The authors compared COPMAN with PEG, ultrafiltration, and Episens-S-based concentration methods [135] and reported a higher sensitivity and recovery compared to other methods in SARSCoV-2-spiked wastewater samples (25.2% (1.0 × 10^3^ copies/mL of HI-SARS-CoV-2) [136]. Furthermore, this method helps recover viruses from liquid and solid fractions, thereby covering a wide range of targets. A direct capture method using silica-based columns (pure yield) for concentration followed by the purification of nucleic acid was proposed by Mondel et al. (2021) [137]. The result showed a 20-fold increase in SARS-CoV-2 RNA when compared with classical PEG/NaCl concentration. Peinado et al. (2022) [138] used a pretreatment step with glycine buffer and showed an increased sensitivity and recovery of control virus (MGV) using a centricon-based ultrafiltration and using adsorption–precipitation (aluminum hydroxide). An automated nucleic acid concentration protocol using magnetic beads in a Kingfisher flex system was reported by Karthikeyan et al. (2021) [139]. The authors reported a 19% higher recovery than PEG and a 14% higher recovery than the electronegative filtration system. The higher recovery was due to the sensitivity of the magnetic bead (Ceres Biotech, Inc., Manassas, VA, USA.), and the automated system eliminated handling discrepancies between samples. Understanding the sample chemistry and target partitioning in wastewater helps in determining the ideal concentration method. The time required to process, resources required, process complications, and labor intensiveness are to be considered as wastewater surveillance involves high-volume sample processing. None of the methods are capable of 100% recovery, suggesting a process investigation to identify the unforeseen loss during sample processing.

#### 1.2.3. Nucleic Acid Extraction from Wastewater Sample

Several commercial nucleic acid extraction systems have been used for wastewater samples. The following section outlines a few significant attempts and comments on their suitability for large-scale operations. A cost-effective direct capture of SARS-CoV-2 from wastewater was proposed by Oscar. N. Whitney (2021) [140], using silica, salt, sewage, and SARS-CoV-2 (4S). The method uses an in-house developed lysis procedure using NaCl and EDTA and extraction using a silica column. This method can be used to extract other fecal viruses (PMMoV), and the extract is stable during storage. The author reports a six-fold increase in yield compared to the ultrafiltration method and is comparatively economical to perform at 13 USD per sample. O’Brien et al. (2021) [141] compared four commercial RNA extraction kits for wastewater collected from a small college population. The Qiagen PowerViral DNA/RNA kit, New England BioLabs Monarch RNA Miniprep Kit, ZymoZymo Quick RNA-Viral Kit, and Quick-RNA Fecal/Soil Microbe Micro Kit were used for comparisons. This study revealed that the Zymo Quick-RNA fecal/soil microbes are effective in extracting RNA from the sample. The author advocates that the use of the preservatives and the specificity of the kit contributed to a higher yield. This approach of concentration followed by purification is time-consuming and laborious in terms of the precipitation–centrifugation of large volumes of samples. Boehm A.B. et al. (2023) [142] used the Chemagic Viral DNA/RNA 300 kit H96 for the Perkin Elmer Chemagic 36.0; the author used a solid fraction of the wastewater sample and reported SARS-CoV-2, influenza A and B virus, RSV, Mpox virus, HMPV, NoV GII, and pepper mild mottle virus. The author performed a PCR inhibitor removal step using the Zymo OneStep-96 PCR Inhibitor Removal kit [97]. In another study, Pérez-Cataluña et al. (2021) [143] compared manual spin column-based extraction with magnetic silica bead-based extraction using the Maxwell RSC Instrument in wastewater seeded with gamma-radiated SARS-CoV-2, porcine epidemic diarrhea virus (PEDV), and mengovirus (MgV). This study showed that aluminum-based precipitation followed by extraction using magnetic beads delivered acceptable sensitivity and reproducibility. Similarly, Qiu et al. (2022) [144] compared five widely used commercially available RNA extraction kits (Qiagen, Venlo, The Netherlands, Thermo Fisher, Waltham, MA, USA, and Promega, Madison, WI, USA) for SARS-CoV-2 extraction from wastewater. Among the chosen kits, MagMAX-96 viral RNA extraction kits using the Kingfisher Flex automated system showed the highest recovery [139].

Although several attempts have been made to establish a standard method for the extraction of RNA/DNA from wastewater, defining a single universal method may not be appropriate because of the diverse nature of wastewater. A myriad of parameters must be considered to establish a method suitable for the regional wastewater. The decision about which method to adopt thus depends on the use case for wastewater testing and resource availability, sensitivity, operational feasibility, and scalability.

#### 1.2.4. Process Automation for Wastewater Analysis

The early detection of infectious diseases plays a crucial role in mitigating the disease’s spread. The lead time for early detection is usually less and varies between diseases and/or until the infected individual seeks clinical testing [145]. As a result, wastewater analysis requires a short turnaround time (TAT) and a high sample frequency for the surveillance of any given catchment area. The primary challenges and potential solutions to meet the TAT are highlighted in the following section.

A manual microbiological analysis approach using plating and culturing is not sufficient to meet the short TAT. An alternative approach involves the use of automated robots for plating and molecular diagnostics. Several automated systems, including microbial plating machines [146], liquid handlers, and automated nucleic extraction systems, have been extensively used for WBE. A comparative study on different automated solutions for microbial plating was conducted by Antony Croxatto et al. (2015) [147] on the InoqulA (BD (Becton, Dickinson Kiestra, Becton Drive Frankilin Lakes, NJ, USA)) and Walkaway specimen processing (WASP) systems (Copan) with manual plating using a defined bacterial consortium and a cloudy urine sample. The InoqulA system produced more discrete colonies at higher concentrations and more statistically significant results than the other chosen methods [148]. A detailed study conducted by Culbreath et al. (2021) [149] revealed the benefits of implementing automation systems in four different facilities with different sample loads. About a 13 to 93% increase in productivity and substantial efficiency and cost savings by using automated microbial plating machines was observed. Similarly, several studies were conducted using liquid handlers and automated nucleic acid extraction systems. Karthikeyan et al. (2021) [150] used Kingfisher Flex (Thermo Fisher Scientific, Waltham, MA, USA) robot systems for the concentration and extraction of nucleic acid from wastewater. The customized extraction plates and reagents were prepared using the Eppendorf ep-motion automated liquid handler, which has reduced process fluctuation and time. Banadaki et al. (2023) [151] developed an automated version of the exclusion-based sample preparation (ESP) RNA concentration and extraction method using PIPETMAX (Gilson), compared the manual extraction efficiency with automated extraction, and proved that automated extraction increased the LOD and sensitivity. In another study, Pérez-Cataluña et al. (2021) [143] compared two concentration and two extraction systems (i.e., manual spin column-based extraction and automated magnetic silica-based extraction using Maxwell RSC Instrument) and concluded that no significant effect was observed in the concentration step, whereas automation using Pure Food GMO and an authentication kit (Promega) showed slightly higher sensitivity than manual extraction. In a similar study conducted by Nicholas W. West et al. (2022) [152], a Chemagic™ 360 instrument with a 12-rod head to extract nucleic acid from raw wastewater was used; unlike the standard method, which involves concentration using PEG/ultrafiltration, the sample (45 mL) was centrifuged and the supernatant (10 mL) was used for RNA extraction. Although these systems require significant capital investment, they are cost-advantageous in the long term because they increase throughput and minimize the workforce, leading to a reduction in the cost per sample [149]. All the automation systems discussed above have some unique advantages over others; it is up to the end user to choose one that is more appropriate for their laboratory setup and sample volume.

#### 1.2.5. The Prospects of Wastewater-Based Epidemiology (WBE)

##### Application of Machine Learning for Outbreak Prediction

The value of the WBE can only be comprehended when it relates to the per capita infection and baseline data from normal surveillance settings. Interpreting such a large dataset manually will not be possible as many variables and individual parameters are considered to interpret the results. In recent times, the application of machine learning (ML) and artificial intelligence (AI) in WBE has been explored due to the capability of self-improvement, identifying patterns in data, and making predictions with limited human interventions. The ML model uses statistical analyses of data to train the parameters, thereby helping in the prediction and identification of patterns in the given dataset. Riberio et al. (2020) [153] attempted to forecast the spread of SARS-CoV-2 using a variety of models, ranging from a simple linear regression model to auto-regressive integrated moving average advance (ARIMA) and support vector regression (SVR) models. The author recommends utilizing the SVR model over the other chosen method for accurate forecasting with minimal errors. In a similar study using a random forest approach, Liam Vaughan et al. (2023) [154] explored the difficulties in machine learning and time series forecasting for COVID-19. The datasets were obtained from the W-SPHERE website (Global Water Pathogens Project 2022). The authors emphasize that sample frequency, training set size, population mobility, and the inclusion of an excessive number of characteristics are the main factors that impact prediction accuracy. A transferable artificial neural network (ANN) model was created by Guangming Jiang et al. [155] in 2022 to forecast SARS-CoV-2 transmission and pandemic dynamics using data gathered from Utah, USA. Prevalence and incidence rates may be predicted with accuracy using the ANN model. Similarly, a model known as the susceptible-exposed-infectious-recovered (SEIR) model was established by Christopher S. McMahan et al. [156] in 2021 to aid in the association between the concentration of SARS-CoV-2 gene fragments isolated in wastewater and the number of infected individuals in the catchment region. The proposed model is further validated with clinical data. Although machine learning and AI technologies are viable techniques for disease outbreak prediction, training a model and verifying it using real-world scenarios are extremely challenging since the developed model must handle various uncertainties in the WBE study.

#### 1.2.6. The Financial Aspects of WBE

The average cost of individual SARS-CoV-2 testing was around USD 148 whereas the cost of WBE is around USD 300 (2022 prices) and covers thousands of individuals in the sampling region [157]. Guerrero-Latorre et al. (2022) [158] established a WBE system that processes 46 samples weekly and biweekly from wastewater treatment plants (WWTPs). The author estimated an annual cost of EUR 20,000 for the weekly process of samples from a single WWTP. Similarly, two pilot laboratories in Nepal were the subject of a thorough cost estimation project by NgwiraI et al. in 2022 [159]. In Blantyre, the expected cost per sample was between USD 25 and 74, whereas in Kathmandu, it was between USD 120 and 175. The price is contingent on the quantity of samples processed; a higher sample count results in a lower cost per sample. In another study, to define the screening strategy in the Tokyo Olympic and Paralympic Village in 2021, a more extensive cost study was conducted, which compared two-step screening methods using PCR individual diagnosis, followed by an antigen test for positive cases against wastewater surveillance. The findings revealed that wastewater surveillance was a cost-effective and justifiable choice for monitoring public health [160]. Although the cost of WBE studies is low compared to clinical surveillance, overall, it can be a financial burden in low-income countries. Therefore, cost-effective methods of wastewater surveillance need to be developed.

## 2. Challenges in Processing Wastewater and Predictability of Disease Outbreak

Although WBE serves as a valuable tool for monitoring infectious diseases and outbreaks in the community, it is worth mentioning that there are still some major challenges in sample processing and the ability to predict outbreaks. For example, consider the following challenges faced in sampling.

1. Community-level wastewater surveillance will not capture communities or facilities served by de-centralized wastewater treatment;
2. Precise mapping of the sewer network is required to estimate the infection load in the catchment area;
3. A balance between accessibility, cost, and sensitivity is yet to be derived for wastewater sampling;
4. The effects of temperature and pH on various wastewater matrix are factors to be considered before sampling;
5. A standard protocol for sampling at different scales is yet to be established.

### 2.1. Varying Limit of Detection

6. Low infection levels in the community may not be captured by wastewater surveillance [161];
7. During the SARS-CoV-2 pandemic, researchers have been able to estimate fecal shedding rates of the virus; however, this has yet to be established for other pathogens.

### 2.2. Hurdles in Correlation with Public Health Data

8. Population dynamics and effects of the floating population affect the accuracy of the WBE;
9. The search for the optimal surrogate to validate pre-treatment procedures has become an additional obstacle. A surrogate that is stable during wastewater processing, has no toxicity, possesses similar structural features to the target pathogen, and is unaffected by the wastewater matrices [162] is needed;
10. The inability of qPCR data to provide data on the virus’s lifecycle represents a significant obstacle. Therefore, it is essential to investigate the survivability of SARS-CoV-2 in wastewater samples and their half-life to comprehend the virus’s transmission between various environmental compartments;
11. Challenges in correlating, sharing, and interpreting routine wastewater surveillance data across different agencies, such as policymakers and public health officials, limit the full potential of wastewater-based epidemiology (WBE);
12. Establishing a link between pathogen detection and the clinical impact or location of cases is often unclear;
13. The lack of updated standardized testing methods poses a challenge, as current approaches are often adapted from clinical diagnostics, which are yet to be validated for wastewater testing.

### 2.3. Complex Wastewater Matrix and Varying Environmental Conditions

14. The complex matrix of urban wastewater containing detergents, xenobiotics, antibiotics, and the presence of PCR inhibitors [70], and varying composition [163], leads to frequent failures and makes the process cumbersome to optimize [164];
15. WBE is not a comprehensive solution for the monitoring of all the circulating pathogens; the limits and drawbacks are to be considered. There is a lack of trained personnel in wastewater surveillance of infectious disease; in most cases, personnel trained in clinical laboratories are utilized for WBE.

## 3. Limitation of This Study

The article attempts to cover the entire process flow of WBE, starting from sampling to the use of advanced methods for the prediction of outbreaks; for this reason, selective studies from the literature were reviewed; the objective of this article is to provide an overview of wastewater-based epidemiological surveillance and its recent trends to the reader. The suggestions provided are based on the reviewed literature and the author’s experience.

## 4. Conclusions

Wastewater surveillance certainly holds the potential to be used as an early warning system and can help in establishing control measures in near-real-time. A variety of barriers that need to be addressed in implementing a WBE system were discussed in the study. An overview of recent advancements in analytical methods and factors to be considered in the interpretation and correlation of the WBE results were presented. The effects of different sampling regimes and storage conditions were compared. A quick summary of pathogens reported in wastewater and the different types of PCR used in quantifying the pathogen were reviewed. Possibilities for integrating an AI-based program for effective monitoring and to create a system that alerts the health authorities were discussed. Further research is needed to assess the impact of WBE in informing policy decisions and guiding public health interventions.

## Figures and Tables

**Table 1:** List of available wastewater surveillance databases.

S. No	Country	Testing Agency	Target Tested	Data/Dashboard Link
1	United State of America	CDC’s National Wastewater Surveillance System (NWSS)	SARS-CoV-2, Respiratory syncytial virus (RSV), Influenza A, Mpox	NWSS Wastewater Monitoring in the U.S. (https://www.cdc.gov/nwss/index.html, accessed on 14 October 2024)
2	Republic of Korea	Department of Analysis of High-Risk Pathogens	SARS-CoV-2	Korea Disease Control and Prevention Agency (https://kdca.go.kr/, accessed on 14 October 2024)
3	Japan	National Institute of Infectious Diseases	SARS-CoV-2	NIJIs—Novel Coronavirus Survey Project in Sewage (https://nijis.jp/#links, accessed on 14 October 2024)
4	New Zealand	ESR (Environmental Science and Research)	SARS-CoV-2	ESR Wastewater Surveillanc (https://esr-cri.shinyapps.io/wastewater/#region=Wellington&log_or_linear=linear&period=twelveMonthsButton, accessed on 14 October 2024)
5	Australia	Government of Western Australia,	SARS-CoV-2 and its variants	COVID-19 wastewater surveillance (https://www.health.wa.gov.au/articles/a_e/coronavirus/covid19-wastewater-surveillance accessed on 14 October 2024)
		New South Wales (NSW), health	Influenza and SARS-CoV-2	NSW respiratory surveillance—COVID-19 and influenza (https://www.health.nsw.gov.au/Infectious/covid-19/Pages/reports.aspx accessed on 14 October 2024)
6	India	CSIR-National Chemical Laboratory (NCL), Symbiosis School of Biological Sciences (SSBS), and the Indian Institute Science Education and Research (IISER) Pune	H1N1, H3N2, Influenza A, and SARS-CoV-2	Wastewater Surveillance Dashboard For Infectious Diseases (COVID-19, H1N1, H3N2, Influenza-A) (https://www.pkc.org.in/pkc-focus-area/health/waste-water-surveillance/wws-covid-dashboard-pune/ accessed on 14 October 2024)
8	Turkey	Turkish Water Institute	SARS-CoV-2	Türkiye Genelinde COVID-19 Yayılımının Atık Sularda SARS-CoV-2 Analizleri ile Takibi (https://covid19.tarimorman.gov.tr/Home/Index accessed on 14 October 2024)
9	Switzerland	ETH, Zurich	SARS-CoV-2, Influenza A, B and RSV	Wastewater Surveillance (https://wise.ethz.ch/ accessed on 14 October 2024)
10	European Union	Digital European Exchange Platform (DEEP)/European Commission’s Health Emergency Preparedness and Response Authority (HERA)	SARS-CoV-2 and Variants of concern	Official SARS-CoV-2 sewage surveillance in the EU (https://wastewater-observatory.jrc.ec.europa.eu/#/dashboards/1/47 accessed on 14 October 2024)
11	Canada	Provincial, territorial, and academic partners across Canada	SARS-CoV-2, Influenza, RSV	Respiratory viruses: Wastewater monitoring dashboard: Respiratory virus activity (https://health-infobase.canada.ca/wastewater/ accessed on 14 October 2024)
12	South Africa	National Institute for communicable Diseases	SARS-CoV-2 and Variants of concern	Wastewater-Based Epidemiology For SARS-CoV-2 In South Africa—NICD (https://www.nicd.ac.za/diseases-a-z-index/disease-index-covid-19/surveillance-reports/weekly-reports/wastewater-based-epidemiology-for-sars-cov-2-in-south-africa/ accessed on 14 October 2024)
13	Global Database-Wastewater SPHERE (SARS Public Health Environmental Response) and COVID poops 19	Data contributed by multiple organizations	SARS-CoV-2	About: Wastewater SPHERE (https://sphere.waterpathogens.org/about accessed on 14 October 2024)

The information was acquired from WHO [30], CDC [31], and from EU Wastewater Observatory for Public Health [32].

**Table 2:** Summary of different sampling methods for detection of pathogen from wastewater.

Sampling	Volume/Duration of Exposure	Pathogen	Detected Concentration/Positive Ratio	Ref.	Remarks
Grab	-	SARS-CoV-2	N1 gene-5.5 log 10 copies/L N2 gene-6.4 log 10 copies/L	[58]	The grab samples, collected between 8 a.m. and 10 a.m., showed less variability than composite sample.
Composite	Varied based on the wastewater flow rate.	SARS-CoV-2	N1 gene-5.3 log 10 copies/L N2 gene-6.3 log 10 copies/L		
Passive sample using cotton buds, electronegative membranes, and medical gauze Composite sampling	24 h 24 h	SARS-CoV-2	25% 41% 31% 50%	[50]	Passive samples made of affordable materials can be used as an economic alternative to expensive auto samplers. The author further used 3D-printed housing units to maintain the mass transfer efficiency.
Composite sampling	1 h	(i) crAssphage (ii) Pepper mild mottle virus (PMMOV) (iii) AdV	log 10 Gene copies/mL (i) 5.95 (ii) 5.08 (iii) 3.93	[57]	The author advocates that 24 h composite samples are likely to be superior to 1 h composite samples.
Composite sampling	24 h	(i) crAssphage (ii) PMMOV (iii) AdV	log 10 Gene copies/mL (i) 5.92 (ii) 5.33 (iii) 3.93		
Passive sampling using i polyethylene-based plastic sampler. ii Cotton-cloth sampler iii Unraveled polypropylene plastic rope sampler.	One week	SARS-CoV-2	Mean gene copies/L (i) 8.493 (ii) 11.909 (iii) 10.358	[49]	The study depicts a positive correlation with composite samples, tested municipal WWTP, and passive samples collected at a city scale.
Passive sampling using tampons	24 h	SARS-CoV-2	Median gene copies/day N2 = 1.29 × 10^9^ N1 = 1.04 × 10^9^	[54]	The author quantified viral RNA by two methods, N1 and N2, and recommends passive sampling approach due to its ease of operation.
Passive sampling using (i) Cheese cloth (ii) Electronegative membrane (iii) Glass wool (iv) Gauze cloth (v) Tampon	24 h	SARS-CoV-2	E gene copies/24 h (i) 4.1 × 10^7^ (ii) 7.3 × 10^6^ (iii) 1.4 × 10^7^ (iv) 1.8 × 10^7^ (v) 2.1 × 10^7^	[56]	The passive sample approach and the medium used was more suitable for other pathogen studies in the work (AdV and Influenza virus).
Composite sample: 24 h composite sample	24 h		E gene copies/24 h 5.4 × 10^7^		

**Table 3:** Sensitivity of different detection methods for WBE.

S. No	Sensitivity	Method Used	Reference
1	2.9–4.6 genome copies/reaction	RT-dPCR	[83]
2	0.066 copies/μL	Reverse transcription-droplet digital PCR (RT-ddPCR)	[84]
3	25 × 10^2^ copies/μL	RT-ddPCR	[85]
4	0.4 copies/μL	Reverse transcriptase loop-mediated isothermal amplification (RT-LAMP)	[86]
5	0.31 × 10^−3^ ng/μL	RT-LAMP	[87]
6	0.0093–9.3 copies/μL	RT-LAMP	[88]
7	10^5^ copies/μL	Nested PCR	[89]
8	1.67 plaque forming units (PFU)	NESTED PCR	[90]
9	7.8 × 10^3^ viruses/liter	NESTED PCR	[10]
10	2.4 × 10^3^ viruses/liter	NESTED PCR	[91]

**Table 4:** Summary of various pathogen detected from wastewater using different PCR and sequencing techniques.

S. No	Targeted Organism	PCR Technique	Sequencing	Country	References
1	SARS-CoV-2	Multiplex PCR	Nanopore sequencing and Illumina sequencing	Netherlands and Belgium	[92,93]
2	SARS-CoV-2	qRT-PCR	Illumina sequencing	United Kingdom	[94]
3	SARS-CoV-2	RT-ddPCR	metatranscriptomic sequencing-Illumina	United States	[95]
4	SARS-CoV-2	qRT-PCR	Sanger Sequencing	United States	[96]
5	SARS-CoV-2	qRT-PCR	-	Netherlands	[27]
6	SARS-CoV-2	dd PCR	-	United States	[97,98]
7	SARS-CoV-2	RT-ddPCR	NextSeq Illumina sequencing	Australia	[99]
8	Rotavirus A (RVA), AstV, NoV, AdV, and SV	RT-PCR	-	Japan	[12]
9	Influenza A and B virus, RSV, Mpox virus, human metapneumovirus (HMPV), NoV GII, and PMMoV	Droplet digital PCR (dd-PCR)	-	Central California, USA	[13]
10	Human AdV, HAV, NoV, and *Salmonella enterica*	Integrated cell culture-PCR (ICC-PCR) and qRT-PCR	-	United States	[100]
11	Enterovirus (EVs), NoV, AdV, Hepatitis A and E virus	qRT-PCR and Cell culture	-	Italy	[101]
12	NoV	RT-PCR	Illumina sequencing	South Africa	[102]
13	Influenza A virus	ddRT-PCR	Sanger Sequencing	United States	[103]
14	Mpox virus	qPCR	Sanger Sequencing	Canada	[104]
15	Mpox virus	qPCR	Sanger Sequencing	Netherlands	[105]
16	*Campylobacter jejuni*	qRT-PCR and Multiplex PCR	16S rRNA	Canada	[106]
17	*C. jejuni* and *C. coli*	qPCR	-	Australia	[62]
18	*Escherichia coli*	qRT-PCR	Illumina sequencing	Germany	[107]
19	*Escherichia coli*	PCR	Illumina sequencing	Czech Republic	[108,109]
20	*E. coli* and *Klebsiella* spp.	PCR	ONT and Illumina sequencing	Switzerland	[110]
21	*Influenza A(H5N1)*	dPCR	-	USA	[111]
22	*Polyomaviruses* (*KI*, *WU*, and *Merkel cell polyomavirus*)	Nested PCR	-	Barcelona	[112]
23	*Salmonella*, *Campylobacter*, and NoV	qPCR and RT-qPCR	-	Oklahoma, USA	[113]
24	*Salmonella*	-	Illumina MiSeq	Hawaii	[114]
